# Online Physician-Patient Interaction and Patient Satisfaction: Empirical Study of the Internet Hospital Service

**DOI:** 10.2196/39089

**Published:** 2023-08-24

**Authors:** Doris Chenguang Wu, Xianduo Zhao, Ji Wu

**Affiliations:** 1 School of Business Sun Yat-Sen University Guangzhou China

**Keywords:** internet hospital, online health service, online physician-patient interaction, patient satisfaction, COVID-19

## Abstract

**Background:**

In China, a form of online health service called the internet hospital became a prominent means of patient care when face-to-face visits were not possible during the COVID-19 pandemic to minimize transmission of the SARS-CoV-2 virus. Patients’ internet hospital experiences largely depend on online physician-patient interaction. Yet, little is known about how physicians can improve patient satisfaction by using specific communication strategies online.

**Objective:**

This study aimed to identify specific communication strategies to help physicians deliver better quality internet hospital services. We also outline recommendations for hospitals to operate internet hospital platforms more effectively.

**Methods:**

A longitudinal data set was collected from an internet hospital platform operated by a top hospital in China. By extracting communication patterns from approximately 20,000 records of online health care services and by controlling the features of service requests, we tested the impacts of response load, more detailed style, and emotional comfort on patient satisfaction. We further explored the effects of these communication patterns in different service contexts.

**Results:**

Physicians with a low response load, a more detailed style, and expressions of emotional comfort received more positive patient feedback. Response load did not affect patient satisfaction with free online health service, whereas a more detailed style and emotional comfort enhanced satisfaction with free service. Response load significantly reduced patient satisfaction with paid online health service, while a more detailed style had no effect. Compared with free service, emotional comfort more strongly promoted patient satisfaction with paid service.

**Conclusions:**

The communication strategies identified can help physicians provide patients with a better internet hospital experience. These strategies require hospitals to schedule each physician’s online service period more appropriately. In addition, tailoring the strategies to service situations can facilitate more targeted and effective internet hospital service for patients.

## Introduction

### Background

The development of internet technology has promoted the use of online health service (OHS) to deliver patient care. OHS covers all health-related actions that hospitals and physicians can provide patients virtually [1,2]. It aims to reduce the burden on the health system and the cost for patients while offering services comparable to in-person appointments [2]. As internet technologies are continuously introduced into the medical industry, OHS is expanding, and such services are performed differently between countries [3]. A form of OHS called “telehealth” has been implemented for many years in developed nations [4,5]. It was first offered through the US Department of Veterans Affairs in the 1950s and has since been applied to mental and physical health care functions, such as online consultation, medical education, and personal training [4]. In China, OHS takes the form of the “internet hospital” and aims to mirror traditional hospital services geared toward physical health. The internet hospital intends to deliver one-stop hospital services, including diagnosis, prescription, and medicine supply, via web technology, electronic prescribing, and medicine e-commerce.

The emergence of COVID-19 forced people to engage in social distancing to reduce the spread of the SARS-CoV-2 virus. Offline health services thus became difficult to access, and the internet hospital quickly rose to prominence as a way to transcend geographical boundaries and serve a large number of patients (patients in remote regions could promptly connect with skilled physicians) [6,7]. The internet hospital has partly alleviated the medical burden in regions hit hard by the outbreak and has facilitated the country’s health management during the pandemic [8]. In the long term, the internet hospital is expected to be an ideal option for the sustainable treatment of postoperative patients, those with chronic diseases, and those with disabilities. The internet hospital can also address problems in traditional hospitals, such as capacity limits, long wait times, and remote health management [8-10]. It further allows for more efficient use of physicians’ free time and can attract new patients through free services to earn future profits. In the long run, the internet hospital will become a mainstream approach of health services and provide public welfare for many patients online. A growing number of scholars have begun to extol its value for patients.

Although the fusion of advanced technologies in the medical and internet fields has elevated the potential of the internet hospital, not all stakeholders believe that this service is comparable to in-person care. The internet hospital operates virtually, such that patients no longer receive tangible service as they would in hospital facilities and environments, which has led to a serious concern regarding patient satisfaction. Patient satisfaction is highly linked with service quality, which is a framework describing the dimensions for patient judgement of the service [11]. Patients have high satisfaction when they are treated in an expected way and receive high service quality. Although the SERVQUAL model is generally used to study customer satisfaction, its fitness for evaluating health care service quality has been doubted [12-14]. Empirical researchers have proposed hierarchical models to measure health care service quality [15]. These models highlight the important dimensions of health care service. Current studies of online health care inherit some important dimensions, such as interpersonal and technical quality [16,17]. Scholars believe that the physician-patient interaction and person-machine interaction are vital to explore in terms of patient satisfaction [11]. Scholars initially pondered the role of information technology adoption in health service, such as the effects of electronic health records [18-20]. However, little is known about how physicians interact with patients virtually. As online physician-patient interaction occurs via text, the parties cannot read one another’s facial expressions or body language. Accordingly, how physicians respond to and communicate with patients is pivotal in this context. Exploring online physician-patient interaction will shed new light on online health service delivery [21].

Few studies have examined interactive strategies to enhance patients’ experiences based on real interactive data, and research on the underlying mechanisms is similarly scarce. Certain characteristics have been shown to moderate the impact of the physician-patient interaction on patient satisfaction [22]. Yet, relevant work has generally pertained to patient characteristics, such as the purpose for using services and the illness severity [23,24]. Little is known about how physicians’ communication characteristics will affect patient satisfaction online. As a complement to prior literature, this study is guided by 2 research objectives. The first is to discover interactive factors that physicians and hospitals should consider to improve patient satisfaction with OHS in the internet hospital. The other is to examine how the relationship between the physician-patient interaction and patient satisfaction with the internet hospital varies in different service contexts.

### Literature Review

Patient satisfaction is an extension of customer satisfaction that describes the extent to which customer expectation is met by suppliers’ services. Previous studies emphasized the relationship between service quality and customer satisfaction [18,25,26]. Yet, few studies have examined the link between patient satisfaction and health care service quality [26]. Patient satisfaction depends on how physicians deliver care service to satisfy patients’ expectations. Patients judge the quality of health service, and when they are benefited as expected or more than expected, they feel satisfied. Similar to customer satisfaction, scores, comments, and thanks during the physician-patient interaction can be important indicators of patient satisfaction [27-30]. Moreover, words of attitude can be used to measure satisfaction in different cultures and contexts. Previous studies measured patient satisfaction from text messages by coding keywords [27,29,30].

The physician-patient interaction is a core component of the physician-patient relationship, which has long been a topic of interest in health services [31]. Research on offline health services has explored the provision of information about diseases or surgeries, prescriptions, and serious diseases in terms of the improvement of patients’ experiences [32-36]. These studies using specific scales for analysis tended to focus on how physicians deliver technical service with regard to expertise and improve the outcome of treatment. In the internet hospital setting, physicians play a key role in cultivating productive physician-patient relationships throughout the diagnosis and treatment processes via real-time text messages. Patients log into the platform having a chat box, type their questions, and receive timely responses from appointed physicians [23]. Unique interactive strategies therefore warrant investigation. Several studies have been conducted on online physician-patient interaction. Table 1 presents a comparison of studies in offline, online, and mobile contexts.

Most of these studies focused on various online situations and proposed different physician-patient interaction measures. Widely used measures include responsiveness, communication strategies, and emotional comfort. Akter et al [41] scaled the impacts of physicians’ willingness to deliver prompt services, confidence, and ability to offer personal care on service quality in a mobile platform [15]. Liang et al [39] considered the following 2 main kinds of interactive strategies: informational support and emotional support, and emphasized their importance in the online health service context. Wu et al [42] further discussed how physicians choose instrumental or affective interaction in a social media situation. In terms of online health communities, Peng et al [43] verified the influence of empathy as an emotional dimension and necessary medical information as an informative dimension, while Yang et al [24] focused on detailed actions of service delivery in the physician-patient interaction and proposed response time, interaction depth, and service content as measures. Furthermore, Tan and Yan [45] pointed out that interaction has scene characteristics and took voice service into consideration.

In summary, these measurements can be roughly grouped into 3 aspects: manner (such as responsiveness, response speed, and interaction depth), communication (such as instrumental interaction, informational support, and service content), and relationship (such as care, empathy, and emotional support), which are consistent with the model by Dagger et al [15]. Dagger et al [15] proposed that interpersonal quality scales include 2 dimensions: interaction and relationship, and the dimension of interaction can be further divided into manner and communication. This study therefore adopted the measure of interpersonal quality from Dagger et al [15] to construct interactions between physicians and patients under the internet hospital context, and 3 aspects of interpersonal quality were considered: manner, communication, and relationship. In particular, due to data availability, we measured *manner* using response load, which represents physicians’ capability of quick and attentive responses to patients’ questions. Response load in this study was measured by the average number of patients a physician serviced per day in the past 1 week. As for *communication*, this study proposed the measure of communication style, quantified by the average length of responses from physicians. Finally, emotional comfort delivered by physicians was considered as the dimension of *relationship*, which was quantified by a dummy variable indicating if physicians comfort patients during the interaction.

**Table 1:** Research on the physician-patient interaction.

Author, year	Factors in the physician-patient interaction	Context
Hadwich et al [37], 2010	Information, trust, individualization, empathy, and ethical conduct	eHealth
Akter and Ray [38], 2010	Responsiveness, assurance, and empathy	Mobile platform
Liang and Scammon [39], 2011	Informational support and emotional support	Online health consultation website
López et al [40], 2012	Interpersonal manner and technical competence	Online ranking website
Akter et al [41], 2013	Cooperation, confidence, and care	Mobile platform
Yang et al [21], 2015	Response speed and interaction frequency	Online health communities
Wu et al [42], 2018	Instrumental interaction and affective interaction	Mobile platform
Yang et al [24], 2019	Response time, interaction depth, and service content	Online health communities
Peng et al [43], 2019	Physicians’ empathy and necessary medical information	Online health communities
Xing et al [44], 2020	Assurance, empathy, reliability, and responsiveness	Online health consultation website
Zhang et al [31], 2018	Interpersonal unfairness and information unfairness	Online health consultation website
Tan and Yan [45], 2020	Informational support, emotional support, response speed, and voice service	Mobile platform
Li et al [7], 2020	Perceived convenience, emotional preference, and perceived information risk	Internet hospital

### Impact of Physicians’ Response Load on Patient Satisfaction

Responsiveness is a central dimension of service quality, especially in service delivery [46]. Responsiveness refers to both the speed of response throughout the physician-patient interaction [47-49] and the extent of attentive and individualized service delivered [50-52]. This concept describes physicians’ approaches of responding to their patients comprehensively. In the internet hospital setting, service delivery is not quite similar to offline service, wherein physicians are on call for online appointments. Physicians can suspend communication and ask patients to wait when they have too many appointments. A heavier response load limits physicians’ attention and attitude toward patients and makes physicians perform indifferently within consulting, especially when physicians are allowed to switch among chat windows of patients. A high response load can even lead to a long break during consulting, because physicians are too busy to treat every patient in time. Therefore, compared with response time, response load can contain more information on physician response. When physicians struggle with many consultations, they give little attention to each patient, and patients can be dissatisfied with physicians’ responsiveness, even though professional advice is provided [53]. Many patients assume that the advice involves copying of stereotypes and believe that their physicians are not taking their concerns seriously [24,45]. We therefore considered physicians’ response load in relation to their responsiveness in the internet hospital, and the hypothesis was as follows:

- H1: Physicians’ response load negatively affects patient satisfaction.

### Impact of Physicians’ Communication Style on Patient Satisfaction

Physicians have different online communication styles, and this may affect patients’ perceived service quality. Therefore, communication style is another fundamental factor in service delivery [45,50]. Moreover, how physicians deliver information to their patients, especially in terms of patients’ conditions, drug use, and surgery, can influence patient satisfaction [41,51-53]. Detailed information from physicians leads to more rich messages in online communication [49]. Moreover, detailed information may be more effective as patients can reread a message at any time in the history record of the chat window [48]. In-depth responses also convey an informative, interpretive, and deliberative communication style, whereas shorter messages suggest a paternalistic style [54]. Some scholars have found that patients expect to receive detailed explanations and a large amount of information from their physicians in the internet hospital [55]. A more detailed style also reflects physicians’ patience and competence, thus assuring patients of their providers’ credibility. We took physicians’ average message length as a proxy of the level of detail in communication, and the hypothesis was as follows:

- H2: Physicians’ more detailed style positively affects patient satisfaction.

### Impact of Physicians’ Emotional Expression on Patient Satisfaction

Sometimes physicians provide emotional support when patients are anxious [46]. In a comfortable interaction, physicians demonstrate their humanity and friendly attitude rather than working as a cold answering machine. Emotional expression and an empathetic attitude are conducive to building trust and good relationships in the physician-patient interaction, ultimately generating greater satisfaction [56]. Patients anticipate receiving assistance with medical information as well as anxiety reduction [57]. Expressions of empathy, such as emotional comfort, imply that physicians care about their patients’ feelings and needs [46,58]. Patients also associate empathy with reassurance and the provision of medical information, and it can boost patients’ optimism in the face of illness and promote trust in the physician-patient relationship [59]. In OHS, physicians should explicitly display empathy to alleviate patients’ mental and emotional discomfort. We therefore put forth the following hypothesis:

- H3: Physicians’ emotional comfort positively affects patient satisfaction.

### Moderating Effect of Free Service

Patients’ personal characteristics are thought to influence their satisfaction with health service delivery [22]. These characteristics also correspond to situational factors such as purchase behavior [21]. Patients’ preferences can influence their satisfaction to varying degrees, even as physicians treat patients similarly. Purchase behavior in the case of the internet hospital involves patients’ selection of either free or paid service, that is, whether patients actually purchase the service or just experience it as a free trial. Unlike that in other countries, online health service in China can be free for 2 reasons. First, in China, online service always begins as a free trial to attract more potential customers and foster their habit of using the platform. As customers get familiar with such a service, the operators push out differentiated pricing services. Second, the Chinese government requires public hospitals to deliver as much free or low-price online medical service as possible to alleviate the pressure on offline health care as well as stabilize the whole society. Therefore, in the internet hospital, patients are allowed to experience a free trial of medical consultation or are required to pay a small amount of money to avail formal medical service. Whether a service is free thus represents an effective dimension to determine how satisfaction differs among patients.

In line with the social exchange theory, patients who choose a paid service will expect physicians to respond more quickly and to provide more support. However, patients selecting a paid service may be less sensitive in terms of their preferred communication style. First, paying patients are already confident in their physicians’ competence, and more detailed information (as a credibility signal) is therefore unlikely to enhance patients’ service satisfaction. Second, patients with a serious illness typically seek immediate and helpful guidance [54]. Based on the above discussion, we hypothesize the following:

- H4: Response load has a stronger influence on patient satisfaction with a paid service than with a free service.
- H5: A more detailed expression style has a stronger influence on patient satisfaction with a free service than with a paid service.
- H6: Emotional comfort has a stronger influence on patient satisfaction with a paid service than with a free service.

**Figure 1:**
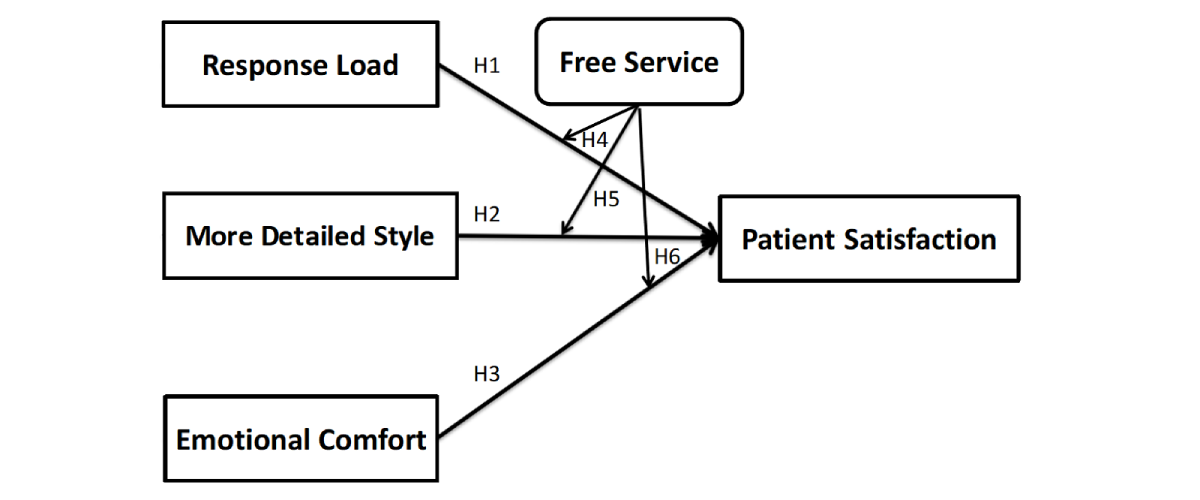
Proposed conceptual model. The hypotheses were as follows: H1, Physicians’ response load negatively affects patient satisfaction; H2, Physicians’ more detailed style positively affects patient satisfaction; H3, Physicians’ emotional comfort positively affects patient satisfaction; H4, Response load has a stronger influence on patient satisfaction with a paid service than with a free service; H5, A more detailed expression style has a stronger influence on patient satisfaction with a free service than with a paid service; H6, Emotional comfort has a stronger influence on patient satisfaction with a paid service than with a free service.

The conceptual model guiding this study has been illustrated in Figure 1.

## Methods

### Data Collection

We collected data from the internet hospital platform of a 3A hospital in China. The data set was appropriate for our hypothesis testing for 3 reasons. First, the data covered nearly 30,000 patient receipts in 2020 across all hospital departments. This volume enabled us to identify common communication strategies based on actual OHS interactions involving various types of illnesses. Second, as the internet hospital has emerged relatively recently in China under the internet economy, these data can facilitate analysis of online physician-patient interaction in the Chinese context. Third, all data were downloaded from the platform’s database and afforded us a direct perspective on the internet hospital. The platform tracks many interaction features that are considered important in online medical consultations. We could therefore control variables irrelevant to our hypotheses to ensure the reliability of our findings.

Our chosen platform was a “mini-program” on WeChat (a popular Chinese social media app) where physicians take turns on duty. Patients can meet with physicians either on demand or by appointment. They upload their personal information, symptom description, and body check in the order list. Once a physician becomes available, patients can type questions in the chat window and receive instant feedback in 12 hours. After 12 hours, only a history record is available for patients. To speed the applicability of the internet hospital and shoulder the social responsibility during the COVID-19 pandemic, the hospital manager decided to offer free service or only one kind of paid service with a charge of around 30 RMB (around 4.14 USD), which is a small amount. The decision was made according to the relevant policy of the Chinese government. To cope with the limited medical services and the medical needs of panicked residents at the beginning of the COVID-19 outbreak, free service was made available. Later, a nominal charge was imposed to avoid wasting manpower and to help those in need when the pandemic was under control. Physicians could be appointed in both kinds of services whose procedures were nearly the same. In our sample, one-third (6745/18,699, 36.07%) of patient receipts involved free service and the remaining (12,954/18,699, 63.93%) involved paid service.

### Ethics Approval

All subjects provided informed consent for inclusion before participation in the study. The study was conducted in accordance with the Institutional Review Board of Sun Yat-Sen University, and the protocol was approved by the Ethics Committee of Sun Yat-Sen University (HS-01705).

### Variable Measures

We focused on the following variables to discern physicians’ behavior during each patient interaction: response load, detailed expression, and emotional comfort. We sought to determine how certain service strategies in OHS influence patient satisfaction. We considered interaction factors as well. Table 2 provides an overview of the dependent variables, independent variables, moderating variables, and control variables in this study.

The variable of patient satisfaction describes patients’ perceptions of their overall experience at the conclusion of the service. In the Chinese context, “thanks” or any similar expression (ie, keywords) at the end of the interaction indicates positive comments and satisfaction toward the physician’s service. Based on the appearance of these kinds of words, we used a dummy variable to describe whether patients expressed appreciation toward their respective physicians as a proxy. We also eliminated situations where patients typed “thanks” as a courtesy in the middle stage of consulting, and such instances only accounted for 1.9% of all interactions in our sample.

The variable of response load describes physicians’ burden to respond to waiting patients. When limited physicians are required to handle both on-demand requests and appointments, their availability to respond to patients can vary, with heavier response loads for physicians leading to longer wait times for patients and formulaic responses within the service. This indicator reflects the degree of responsiveness in the internet hospital. We calculated the average response load for each physician per day on duty as a proxy variable.

The variable of more detailed style describes physicians’ communication style when interacting with patients. Longer text messages from physicians convey an informative, interpretive, and deliberative communication style, whereas shorter text messages embody a more indicative, direct, and paternalistic style. We used the average number of words in physicians’ messages to discern communication styles.

The variable of emotional comfort describes whether physicians use emotional expressions during interactions. In China, physicians commonly use phrases, such as “Don’t worry,” “Don’t be afraid,” and “It’s not a big deal,” to assuage patients’ anxiety. We identified physicians’ comforting actions using a dummy variable based on certain text patterns [60].

Regarding the moderator variable, we used a dummy variable to indicate the service type (1 if the patient selected free service and 0 otherwise). Regarding control variables, we included several dummy variables to control for patients’ geographic characteristics, interaction depth, symptom description length, and disease type.

To ensure the usability and validity of our data, we cleaned the data set by excluding interactions that did not contain any communication record or had missing values for other features (3.58% [1016/28,366] of the total sample). We next identified and removed interactions containing no response from physicians or patients (30.5% [8652/28,366] of the total sample). Then, based on triple standard deviations, we eliminated 182 interactions that included extreme values for our focal variables. The final data set contained 18,709 interactions occurring between January 27, 2020, and December 31, 2020. These interactions involved 14,718 patients from 27 provinces, served by 274 physicians from 36 departments on the chosen internet hospital platform. Three logistic regression models were established to test the respective impacts of response load, more detailed style, and emotional comfort and to identify the moderating effect of free service. We standardized the data before modeling.

**Table 2:** Measurement of study variables.

Variable			Description		Measurement
**Dependent variable**					
	Patient satisfaction	Whether the patient expresses appreciation toward the physician		0: The patient does not thank the physician 1: The patient expresses thanks to the physician during the interaction	
**Independent variable**					
	Response load	The physician’s average patient receipts per week		ln (the number of patients the physician receives per week divided by the number of days the physician is on duty per week)	
	More detailed style	Average word count of the physician’s messages		ln (the total number of words the physician sends in one interaction divided by the total number of messages the physician sends during the interaction)	
	Emotional comfort	Whether the physician comforts the patient		0: The physician does not comfort the patient during the interaction	
**Moderator variable**					
	Free service	Whether the service is free		0: The patient chooses a paid service 1: The patient chooses a free service	
**Control variable**					
	Gender	Patient’s gender		0: Female 1: Male	
	Age	Patient’s age		Number of years	
	Department	Physician’s department		Category variables for 6 major departments	
	Patient residence	Whether the patient lives in Guangdong		0: The patient lives outside Guangdong 1: The patient lives in Guangdong	
	Symptom description	Length of the patient’s symptom description		Shown in the patient’s order before the interaction	
	Interaction frequency	Total number of exchanges during the chat		Shown in the text data of the physician-patient interaction	
	Illness severity	Whether the patient’s illness warrants further treatment and diagnosis offline		0: The physician does not suggest an in-person visit 1: The physician suggests an in-person visit	

## Results

Table 3 presents the descriptive statistics and correlations for the major variables of interest. There was no strong relationship between explanatory variables. The variance inflation factors of variables were all less than 2.0, indicating that multicollinearity was not an issue in our data set.

The results of logistic regression are shown in Table 4. Response load had a significant and negative effect on patient satisfaction (Model 2 in Table 4), that is, patients were unsatisfied when physicians failed to respond promptly and perfunctorily. The relationship between more detailed style and patient satisfaction remained significant and positive when controlling for service content, indicating that patients preferred physicians who provided detailed responses (Model 2 in Table 4). In addition, emotional comfort exerted a positive and significant impact on patient satisfaction (Model 2 in Table 4). Patients appreciated physicians’ comforting words in easing their anxiety. As such, hypotheses H1, H2, and H3 were supported. The moderating effects of free service on the impacts of response load and more detailed style on patient satisfaction were significant and positive (Model 3 in Table 4). Therefore, in terms of paid service, patients were more satisfied with physicians who responded quickly, while in free service, patients preferred to receive longer messages and more professional information from physicians. The moderating effect of emotional comfort was significant and negative, with patients receiving paid service being more satisfied when physicians provided comforting words to relieve their anxiety.

**Figure 2:**
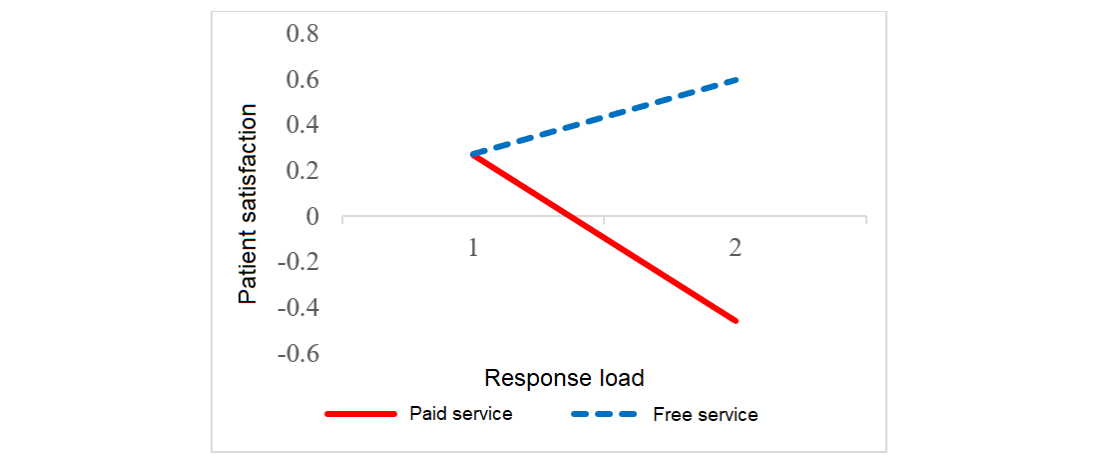
Interaction between free service and response load in influencing patient satisfaction. The solid line represents a significant (*P*<.001) result, and the dashed line represents an insignificant result.

**Figure 3:**
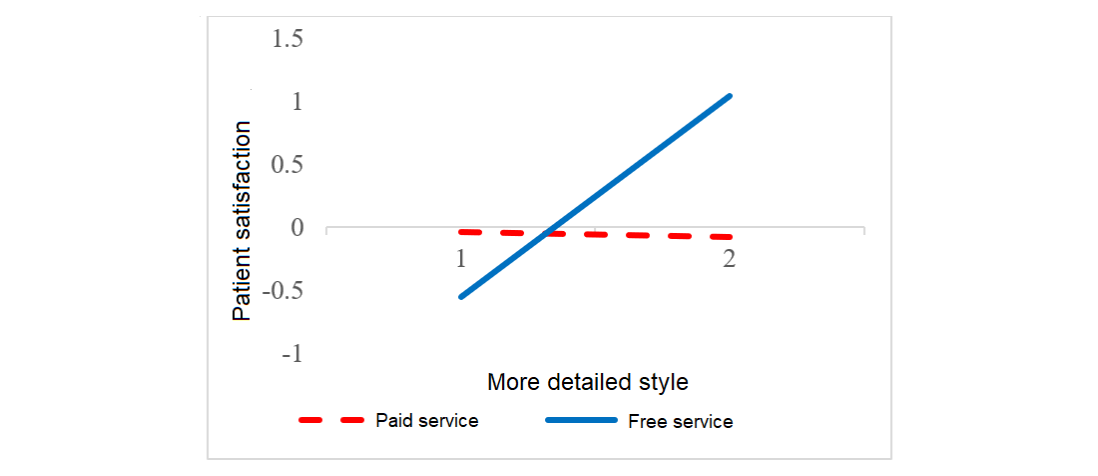
Interaction between free service and detailed expression style in influencing patient satisfaction. The solid line represents a significant (*P*<.001) result, and the dashed line represents an insignificant result.

**Figure 4:**
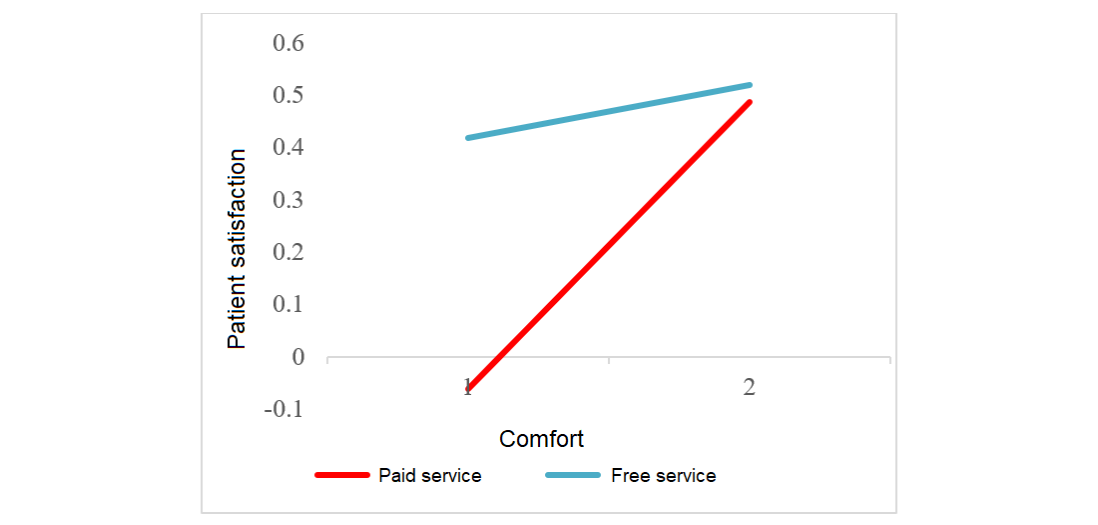
Interaction between free service and emotional comfort in influencing patient satisfaction. The solid line represents a significant (*P*<.001) result.

We tested the moderating hypothesis using interaction effect plots [4]. Figure 2 shows that response load only decreased patient satisfaction with paid service. In other words, only patients receiving paid service expected swifter and more individualized physician responses. Figure 3 depicts that the positive effect of a more detailed style only applied to patients receiving free service (ie, only these patients preferred in-depth communication). Figure 4 indicates that emotional comfort positively influenced patient satisfaction with both free and paid services. Yet, patient satisfaction with paid service rose more rapidly when physicians comforted patients compared with patients receiving free service. Paying for service hence seems to amplify the role of emotional comfort in patient satisfaction.

**Table 3:** Descriptive statistics and correlations of major variables.

Variable	Count	Mean value	SD	Minimum value	Maximum value
Patient satisfaction	18,699	0.49	0.50	0	1
Response load	18,699	38.26	74.31	0.07	302.67
Detailed expression	18,699	101.61	113.51	1	490
Emotional comfort	18,699	0.23	0.42	0	1
Free service	18,699	0.36	0.48	0	1
Gender	18,699	0.41	0.49	0	1
Age	18,699	29.61	10.60	0	94
Neurology department	18,699	0.15	0.35	0	1
Dermatology department	18,699	0.12	0.33	0	1
Traditional Chinese Medicine department	18,699	0.10	0.30	0	1
Respiratory and critical care medicine department	18,699	0.09	0.28	0	1
Endocrinology department	18,699	0.06	0.23	0	1
Endorenal rheumatology department	18,699	0.05	0.23	0	1
Patient’s residence	18,699	0.22	0.41	0	1
Symptom description	18,699	73.98	65.13	10	500
Interaction frequency	18,699	3.07	2.98	1	18
Illness severity	18,699	0.03	0.16	0	1

**Table 4:** Results of logistic hierarchical regression.

Variable			Model 1				Model 2				Model 3		
			Value		*P* value		Value		*P* value		Value		*P* value
**Independent variable^a^**													
	Response load	N/A^b^		N/A		−0.1236^c^		<.001		−0.1715^c^		<.001	
	Detailed expression	N/A		N/A		0.0665^d^		<.05		−0.0081		.82	
	Emotional comfort	N/A		N/A		0.4709^c^		<.001		0.5494^c^		<.001	
**Interaction terms**													
	Free service × response load	N/A		N/A		N/A		N/A		0.2481^c^		<.001	
	Free service × detailed expression	N/A		N/A		N/A		N/A		0.3378^c^		<.001	
	Free service × emotional comfort	N/A		N/A		N/A		N/A		−0.4484^c^		<.001	
**Control variable**													
	Gender	−0.1802^c^		<.001		−0.1830^c^		<.001		−0.1811^c^		<.001	
	Age	0.0479^e^		<.01		0.0457^e^		<.01		0.0487^e^		<.01	
	Neurology department	0.0759		.12		0.1779^e^		<.01		0.2265^c^		<.001	
	Dermatology department	−0.0927		.07		0.0734		.21		0.1830		.77	
	Traditional Chinese Medicine department	0.0908		.11		0.0766		.19		0.0501		.39	
	Respiratory and critical care medicine department	−0.2626^c^		<.001		−0.0104		.89		0.0544		.46	
	Endocrinology department	0.1131		.11		0.1146		.10		0.0792		.26	
	Endorenal rheumatology department	−0.4609^c^		<.001		−0.4550^c^		<.001		−0.4714^c^		<.001	
	Patient’s residence	0.0084		.83		0.0162		.68		0.0255		.51	
	Symptom description	0.2650^c^		<.001		0.2513^c^		<.001		0.2426^c^		<.001	
	Interaction frequency	0.7135^c^		<.001		0.6891^c^		<.001		0.6775^c^		<.001	
	Illness severity	−0.2976^e^		<.01		−0.3768^c^		<.001		−0.3684^c^		<.001	
	Free service	0.0111		.76		0.1071		.07		0.4810^c^		<.001	
Constant			0.1058^e^		<.01		−0.0898^d^		<.05		−0.0622		.12
R square			0.0891		N/A		0.0949		N/A		0.0979		N/A
Observations, n			18,699		N/A		18,679		N/A		18,679		N/A

^a^The dependent variable was patient satisfaction.

^b^N/A: not applicable.

^c^Significant at *P*<.001.

^d^Significant at *P*<.05.

^e^Significant at *P*<.01.

## Discussion

### Principal Findings

This study aimed to examine how the physician-patient interaction affects patient satisfaction in the internet hospital. In the internet hospital, the physician-patient interaction is paramount to patient satisfaction. Delivering a higher quality service and promoting patient satisfaction through better interaction experiences are essential, especially under the high service context of the internet hospital [32,33,61]. Therefore, previous frameworks of service quality cannot be used to accurately describe service delivery in the context of the internet hospital [14,15]. Furthermore, studies on offline communication and interaction skills training have often adopted qualitative methods with small samples as well [62,63]. This study therefore focused on the physician-patient interaction and patient satisfaction under the internet context through big data mining.

As for recent research in the OHS domain, to clarify patients’ perceptions of and experiences with OHS as reported in surveys, subsequent work can lay the foundation for quantitative research comparing online, offline, and mobile care settings [62,64,65]. Although some variables have been proposed to describe the physician-patient interaction, few scholars have examined the internet hospital specifically or addressed the roles of hospitals and physicians in service delivery. We extended knowledge of this domain under the context of the internet hospital, and the work by Dagger et al [15] was used as the theoretical foundation for interaction dimension construction [20,21]. Based on the 3 dimensions of manner, communication, and relationship for interpersonal quality from the work by Dagger et al [15], this study developed the following 3 aspects of the physician-patient interaction under the internet hospital context: response load, detailed communication style, and emotional comfort. A 1-year-long data set from a 3A internet hospital in China was used to examine the impact of these interactions on patient satisfaction, which covered over 10,000 samples of online health care service.

Our results reveal several important themes. In the internet hospital context, hospitals and physicians must devote great effort to adopt decent manners and ensure patient satisfaction. Responsiveness describes such a capability. Rather than merely focusing on the response speed of physicians, we considered responsiveness as physicians’ capabilities to respond with good behavior and attitude under demand pressure [21,41,50-52]. We extracted a series of factors and discovered that, regarding responsiveness, hospitals must provide physicians with manageable schedules and alleviate physician workload [66]. A more reasonable workload can allow physicians to respond timely and allow enough attention to diagnose and treat thoughtfully, communicate with patients in detail, and show good attitude to deliver better service [67].

While the digital platform is being redesigned and improved in a standardized way, scientific and practical methodology to improve physician service quality is the missing piece of internet hospital operation. Compared with previous outcomes, this study highlights the importance of the physician-patient interaction and emphasizes the influences of the interaction on patient satisfaction. Three aspects of the physician-patient interaction were found to significantly affect patient satisfaction: rapid and attentive response; an informative, interpretive, and deliberative communication style; and emotional comfort. These attributes underpin patients’ overall expectations of internet hospital service delivery and foster patient satisfaction. In brief, (1) a lower response load allows physicians to show that they take their patients seriously [3]; (2) a more detailed style implies physicians’ patience and competence; and (3) emotional comfort reflects physicians’ empathy and reliability, boosting trust between patients and physicians [59].

Free service was observed as a situational moderator in the relationships of response load, more detailed style, and emotional comfort with patient satisfaction. During the COVID-19 pandemic, some internet hospitals provided free services. This is the first study to examine the role of free service in physician-patient interaction and patient satisfaction. The findings of this study show that free service could weaken the effects of response load and emotional comfort on patient satisfaction by lowering patient expectation for service quality [19,20]. As for communication style preferences, patients seeking paid service either have already trusted their physicians’ competence or require more direct and instant feedback, and these patients are therefore less concerned about physicians’ message length (ie, longer vs shorter) [24,31,39]. Free service hence increases the impact of a more detailed style on patient satisfaction.

### Limitations

Although we verified our research model and gained valuable insights from this analysis, this study has several limitations, and further investigation is needed in future studies. First, we extracted abstract features of online physician-patient interaction from text data by using statistical methods and text pattern recognition. In this way, although most remarkable features were likely included in our analysis, more complex interaction behaviors in this context may have been omitted. In the future, we expect to extract some other variables that can be discerned in the context by using more powerful data analysis techniques. Second, we explored strategies for physician communication during online interaction in terms of the general perspective, but in reality, there are different communication situations in health services involving an array of communications, such as notifications of critical illnesses, medication reminders, and informed consent forms for surgical procedures. We expect to take a further step to discuss how physicians interact with patients in these types of situations to expand this study. Third, the data we analyzed involved patient and physician samples from a single internet hospital in China. As thousands of internet hospitals are currently in operation, we encourage future research to cover the use of larger samples for better results, which would allow for more extensive and robust conclusions.

### Comparison With Prior Work

There are 3 theoretical contributions of this study. First, to the best of our knowledge, this study is one of the first to empirically explore physicians’ communication strategies in the internet hospital setting. Literature on the physician-patient relationship and patient satisfaction has stressed the importance of physicians’ interactions with patients, especially in OHS. We focused on the internet hospital context to provide a novel perspective on online communication for both hospitals and physicians. Second, we proposed and verified how communication strategies can affect patient satisfaction with OHS. Different from traditional medical services and other OHS platforms, we found that internet hospital physicians have less agency over their response capacity when given heavy workloads. We also applied quantitative methods to examine expression patterns and evaluate physicians’ communication styles based on average message length to determine patients’ internet hospital preferences. Moreover, similar to social support for other patients in online health communities, internet hospital patients need their worries to be addressed. Emotional comfort from physicians can fulfill this need. Overall, we have summarized 3 aspects of online physician-patient interaction. The findings enrich quantitative research on OHS and the internet hospital. Third, dividing patients by service type (ie, free vs paid) allowed us to clarify their expectations and preferences for medical services. We used the social exchange theory to explain patients’ higher expectations about lower response load and emotional comfort when receiving paid service. By testing the moderating hypothesis, we addressed whether patients in different groups had distinct preferences for physician communication. Our work bolsters the understanding of the moderating roles of situational factors in OHS and further contextualizes the variables affecting patient satisfaction.

### Conclusions

This study makes 3 main contributions to contemporary health care practice. First, we underscored the value of the internet hospital as a medical service option. Especially during the COVID-19 pandemic, thousands of internet hospitals assumed some responsibility for public health management. The internet hospital will likely gradually become equivalent to a traditional hospital setting but with unique operations, with patients being able to access medical help more easily, enjoy lower care costs, and avoid the uneven distribution of medical resources in China. The overall efficiency of medical resources and processes will improve.

Furthermore, because the internet hospital model is fairly young, it currently relies on internet technology and smart devices for reconstructing interaction patterns. New guidance for online operations is needed. By extracting factors in online physician-patient interaction, more insights can be gained to inform online hospital operations and communication strategies for physicians. Such strategies can also shape service control and monitoring.

We endeavored to identify variations in patient group expectations for and satisfaction with free and paid services. We recommend that physicians provide patient-oriented service to enhance the perceived competence and competitiveness of hospitals. Tailoring care to patient groups based on backgrounds and situations can better meet their expectations. Patients can later be profiled more precisely to offer highly personalized service.

## Abbreviations

OHS: online health service

## Data Availability

The data are available upon request.
